# ITGAM: A Pivotal Regulator in Macrophage Dynamics and Cardiac Function During Sepsis-Induced Cardiomyopathy

**DOI:** 10.7759/cureus.59342

**Published:** 2024-04-30

**Authors:** Haobin Huang, Qinxue Wang, Luyao Ma, Yanhu Wu

**Affiliations:** 1 Cardiovascular Surgery, The First Affiliated Hospital of Nanjing Medical University, Nanjing, CHN; 2 Geriatrics, The First Affiliated Hospital of Nanjing Medical University, Nanjing, CHN

**Keywords:** immune cell communication, cardiac function, integrin alpha m, macrophage, sepsis-induced cardiomyopathy

## Abstract

Background: Sepsis-induced cardiomyopathy (SIC) is a critical complication arising from sepsis characterized by reversible myocardial dysfunction. Despite the increasing attention to SIC in research, the underlying molecular mechanisms remain poorly comprehended.

Methods: In this study, we utilized bioinformatics to analyze RNA-sequencing (RNA-seq) and single-cell RNA-sequencing (scRNA-seq) data from the Gene Expression Omnibus (GEO) database to identify key immune cell populations and molecular markers associated with SIC. Our experimental approach combined in vitro and in vivo studies to investigate the roles of integrin alpha M (ITGAM) and intracellular adhesion molecule-1 (ICAM-1) in macrophage recruitment and phenotypic polarization, as well as their impact on cardiac function during SIC.

Results: The bioinformatics analysis disclosed significant alterations in gene expression and immune cell composition within the cardiac tissue during SIC, where macrophages emerged as the predominant immune cell type. Notably, ITGAM was identified as a key regulatory molecule that modulates macrophage function, driving the pathogenesis of SIC through its influence on the recruitment and functional reprogramming of these cells. In vitro experiments revealed that lipopolysaccharide (LPS) stimulation triggered an upregulation of ITGAM in macrophages and ICAM-1 in endothelial cells, underscoring their critical roles in immune cell mobilization and intercellular communication. The strategic administration of ITGAM-neutralizing antibodies to SIC mice resulted in a marked decrease in macrophage infiltration within the cardiac tissue, which was initially associated with an improvement in cardiac function. However, this intervention paradoxically resulted in an increased mortality rate during the later phases of SIC, underscoring the complex and dualistic function of ITGAM.

Conclusion: This study provides new insights into the complex dynamics of immune cells within the cardiac environment during SIC, with a particular emphasis on the modulatory role of ITGAM in shaping macrophage behavior. The findings shed light on the reversible nature of myocardial dysfunction in SIC and emphasize the importance of targeted therapeutic strategies for the effective management of SIC.

## Introduction

Sepsis, a life-threatening condition triggered by the body's excessive and dysregulated response to infection, remains a global health challenge with alarmingly high mortality rates [1]. Among the myriad of complications associated with sepsis, sepsis-induced cardiomyopathy (SIC) has emerged as a significant concern [2]. Characterized by reversible myocardial dysfunction and alterations, SIC has garnered considerable attention in both scientific and clinical realms. Nonetheless, deciphering the intricate mechanisms underlying the injury-self-rehabilitation phenomenon still remains challenging [2].

The multifaceted nature of SIC has prompted in-depth studies into its pathophysiology, diagnosis, and management. While definitive evidence remains elusive, abnormalities in global or microcirculatory coronary function are posited as potential causative factors for SIC in some preliminary studies [3,4]. Furthermore, inflammation, through its intricate disturbance involving Toll-like receptor signaling, cytokine production, and inducible nitric oxide synthase activation, precipitates alterations in cardiomyocyte contractility, oxidative stress, and myocardial function in SIC [5,6]. Additionally, mitochondrial dysfunction, stemming from various mechanisms including mitochondrial swelling and mitochondrial DNA damage, plays a crucial role in SIC by disrupting energy production, leading to oxidative stress and potential structural damage [7,8]. While prior studies have elucidated a comprehensive understanding of the molecular mechanisms underpinning SIC, given that SIC is inherently secondary to sepsis, further exploration into the roles of immune cells and pathways modulating cardiac function during sepsis warrants increased attention.

The emerging frontier of genetic lineage tracing and high-dimensional sequencing techniques have provided new opportunities for understanding the roles of different immune cell populations in the pathogenesis of SIC. In this research, we employed comprehensive bioinformatics analysis, utilizing both RNA-sequencing (RNA-seq) and single-cell RNA-sequencing (scRNA-seq) data from the Gene Expression Omnibus (GEO) database, to track immune cell populations and associated molecular markers in SIC. This approach allowed us to identify and characterize the differential expression patterns of integrin alpha M (ITGAM) across various immune cell types within the cardiac microenvironment during SIC. To validate and extend our in silico findings, we complemented these bioinformatics approaches with both in vitro and in vivo experiments. In vitro, we utilized cell line models and primary cells to elucidate the role of ITGAM in macrophage behavior. In vivo, we applied a murine SIC model to assess the therapeutic efficacy of ITGAM neutralization on cardiac function and immune response. With a more profound understanding of the mechanisms underlying SIC pathogenesis, it is anticipated that we can develop increasingly effective, patient-specific therapeutic strategies, augmenting survival prospects while concurrently minimizing adverse effects.

This article was previously posted to the bioRxiv preprint server on February 28, 2024.

## Materials and methods

Data collection for bioinformatics analysis

RNA sequencing (RNA-seq) and single-cell RNA sequencing (scRNA-seq) data pertinent to SIC were acquired from the Gene Expression Omnibus (GEO) database. After detailed retrieval, two datasets, GSE229925 and GSE190856, were selected for further analysis. Both studies employed murine models as the primary research organisms, with the former providing bulk RNA-seq data of the mouse hearts 24 hours after cecum ligation and puncture (CLP) and the latter offering scRNA-seq data of immune cells in mouse hearts at steady state and on days 3, 7, and 21 after CLP. Transcripts per kilobase million (TPM) values were extracted, and genes displaying an average expression level below 0.1 were filtered out. R (v4.3.0) was employed for all bioinformatics analyses.

Bulk RNA-seq data process

We employed the limma package (v3.56.2) for differential gene expression analysis [9]. The adjusted p-value (Padj) was calculated using the Benjamini-Hochberg (BH) method to reduce the false positive rate. The criteria for identifying significantly differential expressed genes were that Padj < 0.05 and |log2FC| > 1 (FC: fold change). Gene Ontology (GO) and Kyoto Encyclopedia of Genes and Genomes (KEGG) pathway enrichment analysis were performed using the R package clusterProfiler (v4.8.3) [10]. Immune cell abundances were estimated using CIBERSORT [11]. Protein-protein interaction (PPI) networks based on the identified differentially expressed genes (DEGs) were constructed using the STRING database [12], and gene pairs with composite scores > 0.7 were imported into the Cytoscape software for further analysis. Hub genes related to SIC were then identified using the cytoHubba plugin in the Cytoscape software [13].

scRNA-seq data process

The GSE190856 scRNA-seq dataset was analyzed using the R package Seurat (v4) [14]. To ensure data quality, we initially performed quality control (QC) by retaining cells with less than 10% mitochondrial gene content and genes expressed in at least three cells within an expression range of 200 to 7,000. We then identified a set of highly variable genes for further study. To address batch effects presented in the data from the different samples, we employed the "Harmony" package. Cell clusters were then generated using the "FindClusters" and "FindNeighbors" functions, and the "t-SNE" method was used to visualize these clusters. Cell annotation was conducted based on the marker genes associated with different cell types, including macrophages (Adgre1, Fcgrt, Timd4, Retnla, Lyve1, Cd163, Folr2, Ccr2, H2-Aa, and H2-Eb1), monocytes (Plac8, Fn1, Ace, Itgal, Napsa, Gngt2, and Chil3), neutrophils (S100a8/a9, Retnlg, Ifitm1, Lcn2, Ngp, and Hp), natural killer (NK)/T cells (Cd3e, Klrk1, Ccl5, Gzma, Il1rl1, Gata3, and Il7r), B cells (Igkc, Ly6d, Ebf1, Cd79b, Ms4a1, and Cd79a), and cycling cells (Mki67, Stmn1, Top2a, Ube2c, and Stmn1). The macrophages were further subdivided into three subsets. The crosstalk among various immune cells was analyzed and then visualized using the R package CellChat [15].

Cell culture and treatment

The mouse monocyte-macrophage leukemia cell line (RAW 264.7) (purchased from the Chinese Academy of Sciences Shanghai Cell Bank, China) was cultivated in Dulbecco's modified Eagle's medium (DMEM) (Gibco, USA) supplemented with 10% fetal bovine serum (FBS) (Gibco, USA), 100 U/mL penicillin, and 100 μg/mL streptomycin (Solarbio, China). The mouse bone marrow-derived macrophages (BMDMs) were extracted from five-week-old male C57BL/6J mice and cultivated in Roswell Park Memorial Institute (RPMI) 1640 medium (Gibco, USA) containing 10% FBS (Gibco, USA), 100 U/mL penicillin, 100 μg/mL streptomycin (Solarbio, China), and 50 ng/mL macrophage-colony stimulating factor (M-CSF) (Novoprotein, China). Mouse cardiac microvascular endothelial cells (MCMECs) (purchased from Procell Life Science & Technology Co., Ltd., China) were cultivated in the complete medium for MCMECs (Procell Life Science & Technology Co., Ltd.). The above cells were cultured in a 37°C atmosphere with 5% CO2.

SIC murine model and treatment

All animal studies were conducted in accordance with ethical standards and were approved by the Institutional Animal Care and Use Committee of Jiangsu Province Hospital. Eight-week-old male C57BL/6J mice were purchased from Jiangsu Huachuang Xinnuo Pharmaceutical Technology Co., Ltd., Taizhou, China, and housed in specific pathogen-free (SPF) facilities with 23 ± 1°C temperature and 12/12 hours light-dark cycle. Lipopolysaccharide (LPS) (Cat# L2630, Sigma, USA) was injected intraperitoneally at a dose of 15 mg/kg to simulate sepsis. CD11b (ITGAM) neutralizing antibody (Cat# BE0007, Bio X Cell, USA) was injected intraperitoneally at a dose of 100 μg per mouse for two consecutive days and one hour prior to LPS modeling. An equal volume of saline was injected into the sham group. Five mice from the LPS group and five from the LPS + neutralizing antibody group underwent echocardiographic examination 24 hours after modeling, followed by euthanasia to collect heart tissues for subsequent assays. Additionally, 10 mice from the LPS group and 10 from the LPS + neutralizing antibody group were continuously monitored up to day 10 to observe the mortality of the model mice throughout the entire course of SIC.

Echocardiography

After isoflurane inhalation anesthesia, transthoracic echocardiography was carried out on mice using the Vevo® 3100 high-resolution microsound equipment (FUJIFILM VisualSonics, Canada). An experienced animal experimenter who was blind to the group assignments performed the ultrasound scan. The left ventricular long-axis section's M-mode pictures were used to record the left ventricular ejection fraction (EF) and fraction of short-axis shortening (FS).

RNA extraction and RT-qPCR

The cardiac tissues, macrophages, and MCMECs were subjected to RNA extraction using the TRIzol reagent (Cat# 15596026, Invitrogen, USA). The HiScript®R II Q RT SuperMix for qPCR (Cat# R222-001, Vazyme, China) was used to synthesize the cDNA, and the RT-qPCR was performed using ChamQ SYBR qPCR Master Mix (High ROX Premixed) (Cat# Q341-02-AA, Vazyme, China) by the LightCycler® 480 System (Roche, Switzerland) according to the manufacturer's instructions. Then, the relative expression of genes was calculated using the 2-ΔΔCT method. The primer sequences are displayed as follows: mouse Itgam Forward (CTTTGGGAACCTCCGACCAG), mouse Itgam Reverse (CACCAAAGTGTCCAAGCCCA), mouse Bnp Forward (GAAGGACCAAGGCCTCACAA), mouse Bnp Reverse (ACTTCAGTGCGTTACAGCCC), mouse Icam-1 Forward (GTGATGCTCAGGTATCCATCCA), mouse Icam-1 Reverse (CACAGTTCTCAAAGCACAGCG), mouse Il-1β Forward (TGCCACCTTTTGACAGTGATG), mouse Il-1β Reverse (TGATGTGCTGCTGCGAGATT), mouse Il-6 Forward (GACAAAGCCAGAGTCCTTCAGA), mouse Il-6 Reverse (TGTGACTCCAGCTTATCTCTTGG), mouse Mcpt-1 Forward (GAGGACAGATGTGGTGGGTT), mouse Mcpt-1 Reverse (AGGAGTCAACTCAGCTTTCTCTT).

Statistical analysis

Statistical analyses were conducted using the GraphPad Prism v8.3.0 software. The normality of the data was assessed using the Shapiro-Wilk test. For data adhering to a normal distribution, comparisons between two groups were performed using an unpaired t-test with Welch's correction. In cases of non-normal distribution, a standard unpaired t-test was utilized. For the analysis of survival data, Kaplan-Meier survival curves were generated, with intergroup differences evaluated using the log-rank (Mantel-Cox) test. All statistical tests were two-tailed, with a p-value threshold of less than 0.05 set for establishing statistical significance.

## Results

Identification of the hub genes related to SIC in the bulk RNA‑seq dataset

**Figure 1:**
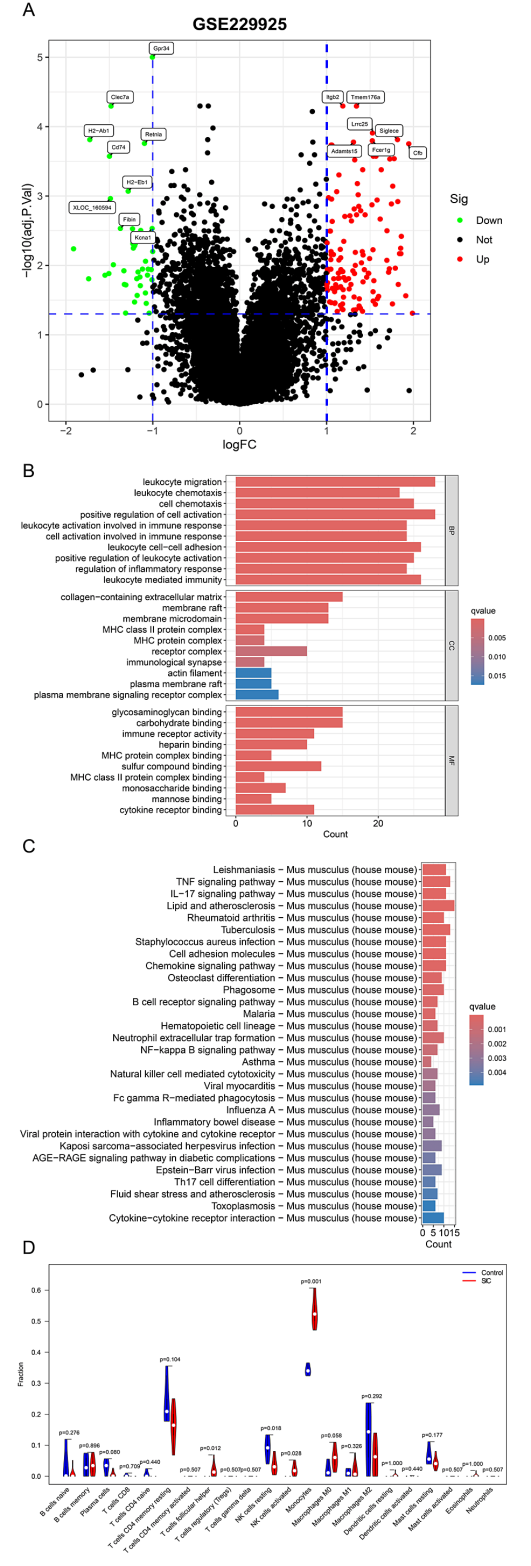
Immune cell infiltration and molecular pathway enrichment in the cardiac tissue of sepsis-induced cardiomyopathy mice A: This volcano plot displays the upregulated and downregulated genes in the CLP group as compared with the sham group. B and C: Enrichment analysis (GO and KEGG) indicated an elevated inflammatory state in the cardiac tissue of SIC mice. D: CIBERSORT analysis showing the comparative immune cell composition in the cardiac tissue of SIC and control mice, with a notable increase in monocyte infiltration during SIC. CLP: cecum ligation and puncture, GO: Gene Ontology, KEGG: Kyoto Encyclopedia of Genes and Genomes, SIC: sepsis-induced cardiomyopathy, MHC: major histocompatibility complex, TNF: tumor necrosis factor, IL-17: interleukin-17, NF-kappa B: nuclear factor-kappa B, AGE-RAGE: advanced glycation end products-receptor for AGEs, NK: natural killer

Upon analysis of the GSE229925 dataset, we identified 149 significantly upregulated genes and 43 significantly downregulated genes in the CLP group when compared with the sham group (Figure 1A). Through enrichment analysis (GO and KEGG), we observed that within the SIC group, pathways associated with the activation of immune cells (e.g., leukocyte migration and cell activation involved in immune response) and the regulation of chemokines (e.g., tumor necrosis factor (TNF) signaling pathway and interleukin (IL)-17 signaling pathway) were significantly enriched, suggesting a higher inflammatory state in the cardiac tissue during SIC (Figure 1B, 1C). We subsequently performed CIBERSORT analysis to unveil the immune cell composition within the cardiac tissue of SIC mice. The result revealed a marked increase in monocyte infiltration in the CLP group compared with the sham group (Figure 1D). Furthermore, a PPI network was constructed using the STRING database and then imported into the Cytoscape software, where 15 hub genes (*Vav1*,* Cxcl1*,* Itgam*,* Selp*,* Icam1*,* Ccl2*,* Cd44*,* Stat3*,* Spi1*,* Itgb2*,* Hp*,* Fos*,* Timp1*,* Lcn2*, and *Sele*) were identified using cytoHubba.

Characterization of hub gene expression within distinct cardiac immune cells during SIC

**Figure 2:**
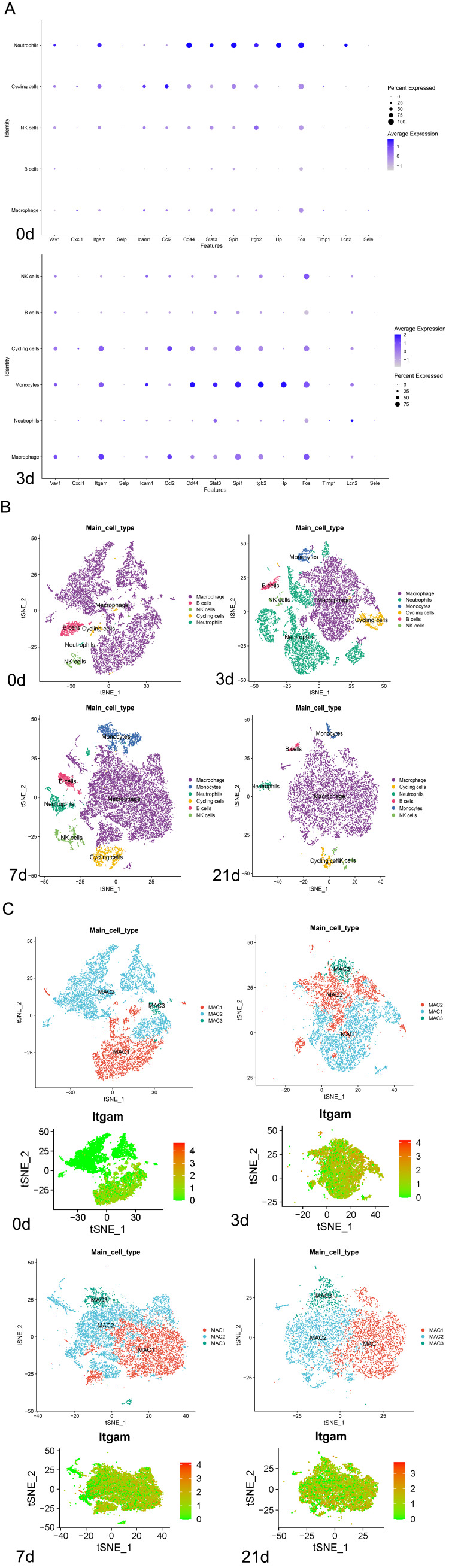
Dynamics of immune cell populations and hub gene expression in SIC A: Expression profiles of hub genes across various cardiac immune cell types at steady state and following CLP. *Itgam* shows a marked increase within macrophages post-CLP. B: Temporal analysis depicting the composition of cardiac immune cells at steady state and on days 3, 7, and 21 post-CLP. A notable decrease in macrophages is observed on day 3, followed by an increase on days 7 and 21. Monocytes and neutrophils exhibit significant infiltration post-CLP, with peaks at different time points. C: Macrophages (MAC1, MAC2, and MAC3) demonstrate varied expression of *Itgam* across different subclusters, with significant upregulation in the MAC2 subcluster post-CLP, indicating its involvement in the pathogenesis of SIC. SIC: sepsis-induced cardiomyopathy, CLP: cecum ligation and puncture, NK: natural killer

We further explore the expression profiles of the 15 hub genes in different cardiac immune cells using the scRNA-seq data (GSE190856). Single-cell transcriptomic profiles of mouse hearts at steady state and on days 3, 7, and 21 following CLP were separately analyzed. Cardiac immune cells were categorized into six primary clusters, namely, macrophages, monocytes, neutrophils, natural killer (NK)/T cells, B cells, and cycling cells. Macrophages emerged as the most prevalent immune cell type within the cardiac environment. Among the 15 hub genes, *Itgam*, widely acknowledged as a signature marker of macrophages, demonstrated a notable upsurge in expression within macrophages after CLP (Figure 2A).

Our temporal analysis of immune cell composition post-CLP revealed an initial decline in the macrophage population on day 3, with a subsequent rebound and increase on days 7 and 21. Monocyte was negligible at steady state but showed a significant influx on day 3 post-CLP, peaking on day 7 before gradually receding by day 21. Additionally, a pronounced surge in neutrophil infiltration was detected on day 3 post-CLP (Figure 2B).

Given that cardiac macrophages can be classified into various subtypes according to their molecular markers, functional roles, and embryonic origins, we proceeded to examine the variability in *Itgam* expression across distinct macrophage subclusters. Cardiac macrophages in the GSE190856 dataset were categorized into three distinct subclusters (MAC1, MAC2, and MAC3). MAC1 was characterized by high expression of *Timd4*, *Retnla*,* Lyve1*, *Cd163*, and *Folr2*, representing the self-renewing TLF^+^ resident macrophage subtype in the myocardium [16,17]. MAC2 corresponded to the MHC-II^hi^ macrophages; they can be partially supplemented by circulating monocytes but do not undergo continuous renewal [16,17]. MAC3 represented the CCR2^+^ macrophages; they also expressed high levels of antigen presentation genes (H2-Aa and H2-Eb1) and could be rapidly replenished by monocytes [16-18]. At steady state, *Itgam* expression was predominantly observed in the MAC1 and MAC3 subclusters. Post-CLP, however, a significant upregulation of *Itgam* in the MAC2 subcluster was observed, implicating an association between increased *Itgam *expression in the MAC2 subcluster and the pathogenesis of SIC (Figure 2C).

ITGAM-mediated immune cell communication during SIC

**Figure 3:**
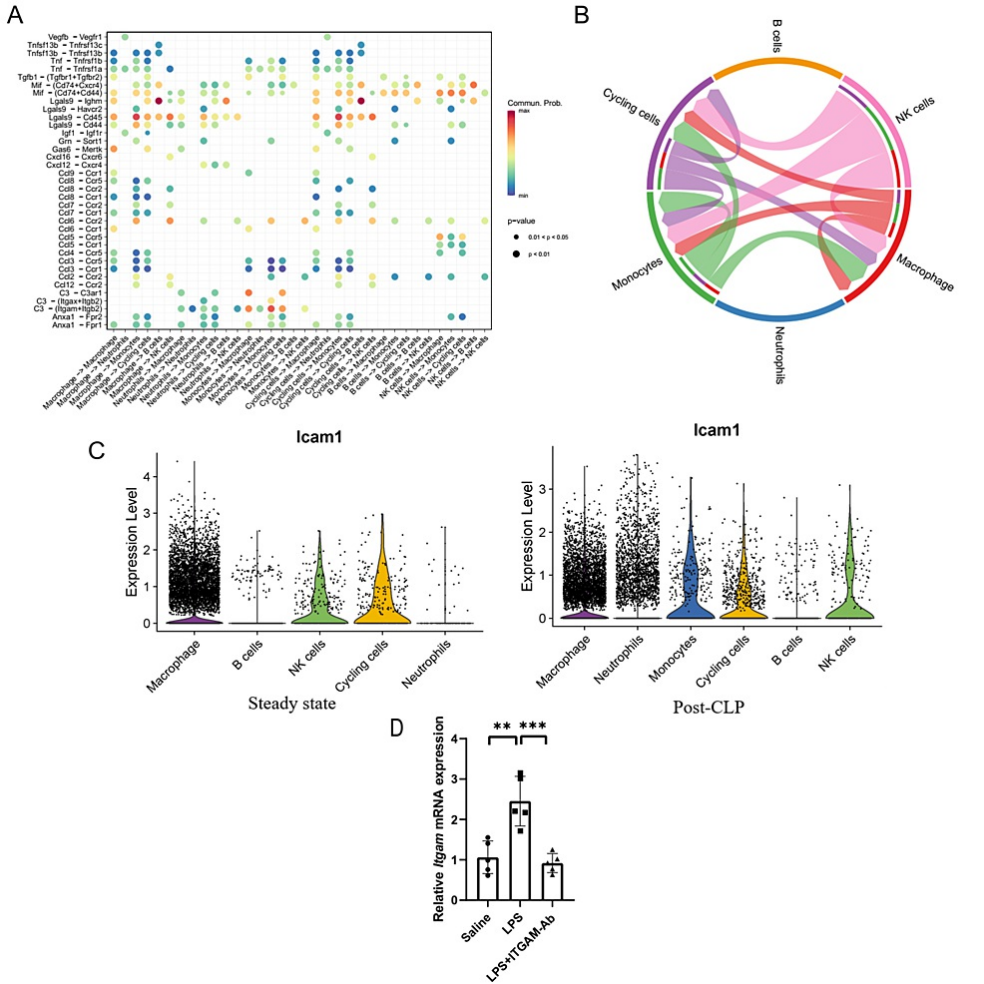
Analysis of ITGAM and ICAM-1 dynamics in cardiac immune cell communication during SIC A: Activation of the C3 - (Itgam+Itgb2) ligand-receptor pair post-CLP, highlighting enhanced signaling interactions particularly from monocytes to various immune cells including macrophages, monocytes, and cycling cells, underlining the critical role of ITGAM in mediating immune cell communication during SIC. B: Cell-cell contact communication facilitated by the Icam1 - (Itgam+Itgb2) ligand-receptor pair, predominantly involving monocytes, macrophages, NK cells, and cycling cells. C: Analysis of *Icam1* expression across various immune cell types at steady state and following CLP. D: Graphical representation of the pronounced elevation in *Icam1* expression within myocardial tissues post-CLP, as derived from the GSE229925 dataset. ITGAM: integrin alpha M, ICAM-1: intracellular adhesion molecule-1, SIC: sepsis-induced cardiomyopathy, CLP: cecum ligation and puncture, NK: natural killer

As ITGAM typically combines with the integrin subunit beta 2 (ITGB2) to form a leukocyte-specific adhesion receptor also known as macrophage receptor 1 (Mac-1), we further investigated the role of ITGAM in facilitating communication among diverse immune cells by using the "Secreted Signaling" and "Cell-Cell contact" databases curated in CellChat. Employing the "Secreted Signaling" database revealed significant activation of the C3 - (Itgam+Itgb2) ligand-receptor pair after CLP, thereby contributing to the signaling from monocytes to macrophages, monocytes, and cycling cells (Figure 3A). On the other hand, when using the "Cell-Cell contact" database, we found that the ICAM signaling, which was known for regulating leukocyte recruitment from circulation to sites of inflammation, was upregulated through the Icam1 - (Itgam+Itgb2) ligand-receptor pair. Notably, we identified that monocytes, macrophages, NK cells, and cycling cells were the primary immune cell types engaging in cell-cell contact communication via this ligand-receptor interaction (Figure 3B).

Similar to* Itgam*, *Icam1* was also identified as one of the 15 hub genes associated with SIC in this study. We then examined its expression in distinct cardiac immune cells post-CLP. However, unlike *Itgam*, *Icam1* did not exhibit a comparable level of upregulation in these cells (Figure 3C). To comprehensively evaluate the global expression profile of the *Icam1* gene in cardiac tissues during SIC, the GSE229925 dataset was utilized, and the analytical outcomes revealed a pronounced elevation in* Icam1* expression within the myocardial tissues post-CLP (Figure 3D). We thus hypothesize that the heightened *Icam1* expression is predominantly localized to non-immune cells, such as endothelial cells, during the development of SIC.

Upregulation of *Itgam* in macrophages and *Icam1* in endothelial cells following LPS stimulation

**Figure 4:**
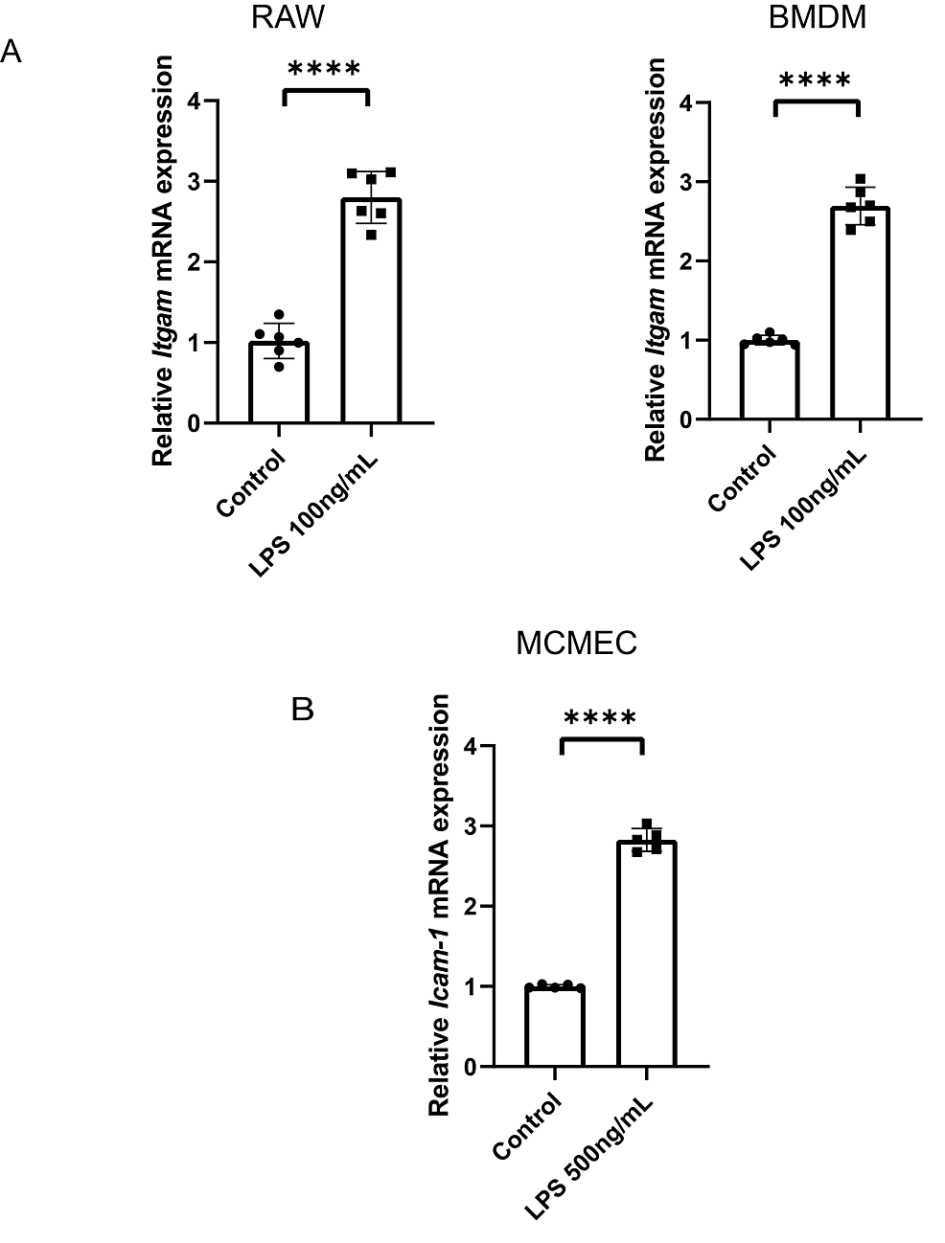
Upregulation of Itgam in macrophages and Icam1 in endothelial cells following LPS stimulation A: Quantitative PCR analysis shows significant upregulation of *Itgam* mRNA expression following LPS stimulation in both RAW 264.7 mouse monocyte-macrophage leukemia cell lines and primary mouse BMDM, highlighting the responsive nature of macrophages to septic stimuli. B: The relative *Icam1 *mRNA expression in MCMEC after LPS stimulation. The marked elevation in *Icam1* expression upon exposure to LPS (500 ng/mL) underscores the responsiveness of endothelial cells in the context of SIC. LPS: lipopolysaccharide, PCR: polymerase chain reaction, mRNA: messenger RNA, BMDM: bone marrow-derived macrophages, MCMEC: mouse cardiac microvascular endothelial cells, SIC: sepsis-induced cardiomyopathy

We utilized LPS to stimulate the RAW 264.7 mouse monocyte-macrophage leukemia cell line, as well as primary mouse bone marrow-derived macrophages (BMDM), aiming to replicate the septic state in an in vitro setting. Subsequent qPCR revealed a notable elevation in *Itgam* expression in both RAW 264.7 cells and BMDM following LPS stimulation (Figure 4A). To further verify the hypothesis of* Icam1* upregulation in endothelial cells during SIC, mouse cardiac microvascular endothelial cells (MCMEC) were subjected to LPS stimulation, resulting in a marked increase in *Icam1* expression (Figure 4B).

Impact of ITGAM neutralization on macrophage recruitment and cardiac outcomes during SIC

**Figure 5:**
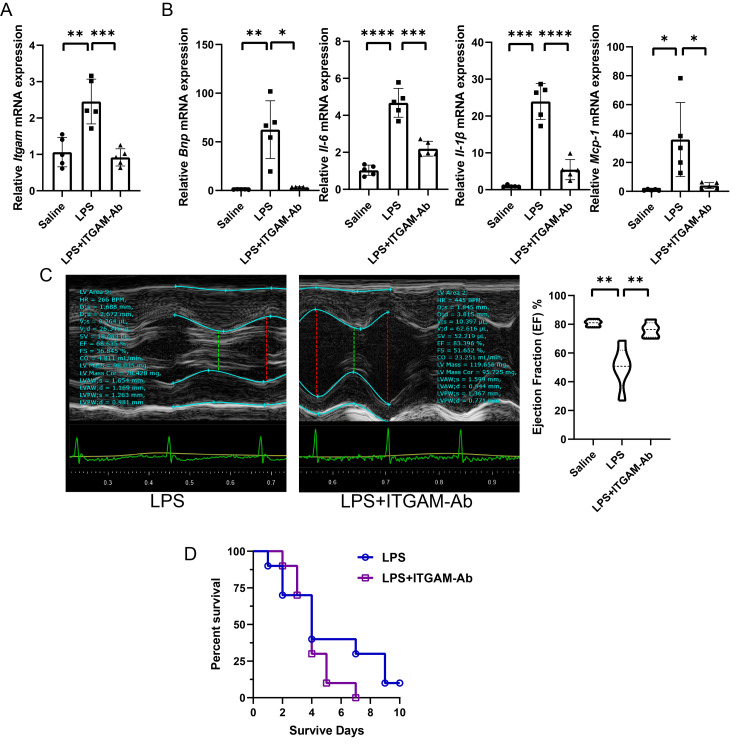
Impact of ITGAM neutralization on macrophage recruitment, inflammatory response, cardiac function, and survival in SIC A: The relative expression of *Itgam* mRNA in myocardial tissues following the administration of ITGAM-specific neutralizing antibody. B: The relative mRNA expression levels of the heart failure marker* Bnp* and inflammation-related genes *Il-6*,* Il-1β*, and *Mcp-1 *in the myocardial tissues. Notably lower* Bnp *mRNA expression in ITGAM-Ab-treated mice indicates reduced heart failure. Similarly, reduced expression of inflammatory genes *Il-6*, *Il-1β*, and *Mcp-1* underscores a diminished inflammatory response in the heart following ITGAM neutralization. C: Echocardiography showing improved cardiac function in ITGAM-Ab-treated mice compared to those receiving only saline. This finding highlights the cardioprotective effect of ITGAM inhibition during the early phase of SIC. D: Survival curves over a 10-day period post-LPS challenge for mice treated with saline or ITGAM-Ab. Despite the early cardioprotective effects, the overall mortality rate was higher in the ITGAM-Ab group, with the majority of deaths occurring in the mid to late stages of SIC, reflecting the complex role of ITGAM in the disease's pathophysiology. ITGAM: integrin alpha M, SIC: sepsis-induced cardiomyopathy, mRNA: messenger RNA, Ab: antibody, LPS: lipopolysaccharide

To further elucidate the role of ITGAM in SIC, we administered an ITGAM-specific neutralizing antibody to mice to inhibit ITGAM function in vivo before giving the LPS intraperitoneal injection for modeling. Despite the administration of an ITGAM-specific neutralizing antibody not directly reducing* Itgam* expression, we indeed observed a decrease in* Itgam* expression within the heart during early SIC. This reduction in expression is probably attributed to the diminished infiltration of ITGAM-positive monocytes/macrophages into the heart tissue. As previously established, SIC is characterized by an initial decrease in cardiac macrophages. In the absence of ITGAM neutralization, circulating ITGAM-positive monocytes/macrophages would be recruited to the heart via ICAM-1 on endothelial cells, thereby replenishing the macrophage population. By neutralizing ITGAM, we effectively curtailed this recruitment process. This effect was paralleled by lower expression of Bnp mRNA, a heart failure marker, in the neutralizing antibody-treated group compared to controls 24 hours post-LPS challenge. Additionally, there was a notable decrease in the expression of inflammation-related genes in the neutralizing antibody-treated group, including *Il-6*, *Il-1β*, and *Mcpt-1*, suggesting a reduction in inflammatory responses within the heart (Figure 5B). Echoing these molecular findings, small animal echocardiography revealed a cardioprotective effect of ITGAM inhibition in the early phase of SIC, as evidenced by improved cardiac function (Figure 5C). However, despite these early benefits, the overall mortality rate was higher in the neutralizing antibody group throughout the disease course, with the majority of deaths occurring in the mid to late stages of SIC (Figure 5D), highlighting the complexity of ITGAM's role in the pathophysiology of SIC.

## Discussion

In this study, we have delved deep into the dynamic landscape of gene expression and immune cell dynamics within cardiac tissues afflicted by SIC. Our comprehensive analysis has shed light on the pivotal role of macrophages, marked by the upregulation of ITGAM, in the cardiac tissue during SIC. This study has also underscored the importance of ITGAM and ICAM-1 in the modulation of immune cell interactions, which is crucial for understanding the molecular underpinnings of SIC. These insights are fundamental not only for advancing our knowledge of SIC but also for paving the way toward the development of targeted therapeutic strategies.

The resident cardiac macrophages (RCMs) are recognized for their protective functions in maintaining cardiac integrity and function under various pathological conditions, including myocardial infarction, heart failure, and myocarditis [16]. In the context of SIC, TREM2^hi ^RCMs scavenge cardiomyocyte-ejected dysfunctional mitochondria, thus preventing excessive inflammation and sustaining normal cardiac function [18]. We have further classified macrophages into three distinct subtypes based on their molecular markers and functional roles: the self-renewing MAC1, the MHC-II^hi^ MAC2, and the CCR2^+^ MAC3 [17,18]. The significant upregulation of *Itgam* in MAC2 post-CLP indicates a dynamic shift in this subtype's role during SIC progression.

ITGAM, also known as CD11b, synergizes with the beta 2 chain (ITGB2) to constitute a leukocyte-specific integrin, commonly recognized as Mac-1. This integrin is documented as essential for facilitating the adhesion and transmigration of neutrophils and monocytes to activated endothelium, primarily through ICAM-1 on the activated endothelial cells, thereby directing these immune cells to migrate toward sites of infection or inflammation [19-22]. Upon further examination, we did discover that LPS stimulation not only upregulated *Itgam* in peripheral macrophages (using the RAW cell line and BMDM) but also induced a significant rise in *Icam1 *expression in endothelial cells (using MCMEC), hinting at a complex recruitment dance orchestrated by these markers in the heart during SIC. As it has been observed that the monocytes infiltrating the heart gradually adopt the transcriptional profiles of RCMs [17], we supposed that the recruited monocytes/macrophages acquired the phenotypic characteristics of MAC2, thus making the MAC2 also exhibit high levels of *Itgam* expression during SIC.

**Figure 6:**
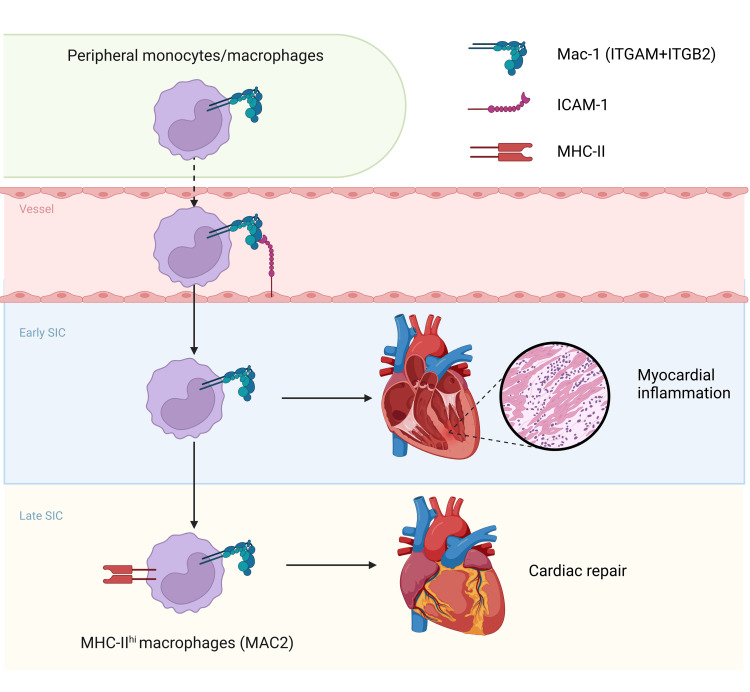
Mechanistic diagram illustrating the role of ITGAM in SIC Early in SIC, the interaction between Mac-1 (ITGAM+ITGB2) on peripheral monocytes/macrophages and ICAM-1 on endothelial cells facilitates the adhesion and migration of monocytes/macrophages to the inflamed myocardial tissue, thus exacerbating the inflammatory response within the heart, intensifying myocardial damage. As SIC progresses to a later stage, these cells transform into MHC-II^hi^ macrophages (MAC2) and participate in the cardiac repair process. This diagram highlights the dual roles of ITGAM in promoting inflammation and facilitating repair within the cardiac microenvironment across different phases of SIC. ITGAM: integrin alpha M, SIC: sepsis-induced cardiomyopathy, ICAM-1: intracellular adhesion molecule-1, MHC: major histocompatibility complex This diagram was created with BioRender.com.

MHC-II^hi^ macrophages (MAC2), known primarily for antigen presentation, are increasingly recognized for their immunoregulatory and cardioprotective roles, particularly relevant in cardiac stress conditions such as pressure overload and myocardial infarction [23,24]. These properties suggest their potential as mediators of disease resolution in the latter stages of SIC. Furthermore, Mac-1, an integrin whose expression significantly increases in MAC2 during SIC, embodies a dual function in both promoting and mitigating inflammation [21]. Studies have highlighted its critical role in causing neutrophil recruitment, endothelial injury, and thrombosis, while also contributing to anti-inflammatory processes and maintaining endothelial integrity [25]. Drawing parallels from previous findings, our study suggests that during SIC, monocytes/macrophages recruited to the heart not only exacerbate the inflammatory milieu at the early stages of SIC but, upon phenotypic transformation to MAC2, potentially initiate a shift toward an immunoregulatory and reparative phase, hinting at a multifaceted role of these cells in SIC progression (Figure 6).

Despite the comprehensive nature of our findings in elucidating the mechanisms and cellular dynamics of SIC, this study is not without its limitations. Firstly, our reliance on bioinformatics analysis of specific datasets, which exclusively comprise data derived from murine models, while providing a robust foundation for our hypotheses, also means that our conclusions are inherently tied to the limitations of these datasets. This murine focus may affect the generalizability of our findings to human conditions. The extrapolation of these findings to broader contexts should be approached with caution. Furthermore, while our study offers valuable insights into the role of ITGAM and ICAM-1 in macrophage recruitment and cardiac outcomes during SIC, it primarily focuses on the early stages of the disease. The higher mortality observed in the neutralizing antibody group in the later stages of SIC suggests complex underlying mechanisms that our study did not fully explore. This indicates a need for more extensive experimental studies to understand the long-term effects of ITGAM inhibition and the intricate balance between protective and detrimental immune responses in SIC.

## Conclusions

This research elucidates the intricate involvement of ITGAM in the development of SIC, emphasizing its biphasic impact on myocardial function. Our findings suggest that ITGAM-mediated recruitment of circulating monocytes/macrophages in early SIC amplifies cardiac inflammation, leading to impaired cardiac function. Yet, as SIC evolves, these infiltrating cells transform into ITGAM^+^ MAC2, potentially mitigating adverse effects and facilitating cardiac recovery. Our findings provide crucial insights into SIC's molecular underpinnings and emphasize the importance of further research to translate these murine model observations into human clinical applications, aiming to refine SIC treatment strategies.
